# A multicenter retrospective study of PD-1 blockade plus chemotherapy as first-line therapy in advanced hepatoid adenocarcinoma of the stomach

**DOI:** 10.1093/oncolo/oyaf312

**Published:** 2025-09-25

**Authors:** Jingru Wang, Jinbo Zhan, Zhen Rao, Gang Su, Yan He, Ling Zhou, Jianhua Wu, Xiaowei Sun, Xiaojun Xiang

**Affiliations:** Department of Oncology, the First Affiliated Hospital, Jiangxi Medical College, Nanchang University, Nanchang, Jiangxi 330000, P.R.China; Department of Oncology, the First Affiliated Hospital, Jiangxi Medical College, Nanchang University, Nanchang, Jiangxi 330000, P.R.China; Department of Gastric Surgery, Sun Yat-sen University Cancer Center, State Key Laboratory of Oncology in South China, Collaborative Innovation Center for Cancer Medicine, Guangzhou, Guangdong 510000, P.R.China; Department of Cardiac Vascular Surgery, The First Affiliated Hospital of Soochow University, Suzhou Medical College, Soochow University, Suzhou, Jiangsu 215000, P.R.China; Department of Oncology, the First Affiliated Hospital, Jiangxi Medical College, Nanchang University, Nanchang, Jiangxi 330000, P.R.China; Department of Oncology, the First Affiliated Hospital, Jiangxi Medical College, Nanchang University, Nanchang, Jiangxi 330000, P.R.China; Department of Oncology, Nanfang Hospital, Southern Medical University, Guangzhou, Guangdong 510515, P.R.China; Department of Gastric Surgery, Sun Yat-sen University Cancer Center, State Key Laboratory of Oncology in South China, Collaborative Innovation Center for Cancer Medicine, Guangzhou, Guangdong 510000, P.R.China; Department of Oncology, the First Affiliated Hospital, Jiangxi Medical College, Nanchang University, Nanchang, Jiangxi 330000, P.R.China; Department of the Rare Disease Center, the First Affiliated Hospital, Jiangxi Medical College, Nanchang University, Nanchang, Jiangxi 330000, P.R.China

**Keywords:** hepatoid adenocarcinoma of the stomach, PD-1 blockade, immunotherapy, biomarker

## Abstract

**Purpose:**

This study aims to evaluate the efficacy and safety of programmed cell death protein 1 (PD-1) blockade in combination with chemotherapy for patients with advanced hepatoid adenocarcinoma of the stomach (HAS).

**Materials and methods:**

This study retrospectively collected data from 25 patients with advanced HAS who received first-line PD-1 blockade combined with chemotherapy across 6 centers between January 2018 and January 2024. Progression-free survival and overall survival were assessed using Kaplan-Meier curves.

**Results:**

This study included 25 patients with HAS, all of whom received a first-line treatment regimen combining PD-1 blockade and chemotherapy. The objective response rate and disease control rate were 76.0% and 88.0%, respectively. The median follow-up time was 13.1 months, with a median progression-free survival of 10.2 months (95% CI, 6.3-14.1) and a median overall survival of 20.3 months (95% CI, 11.3-29.4). A total of 20 patients (80.0%) experienced adverse reactions of varying degrees, with white blood cell count decreased (12, 48.0%) being the most common adverse event. Two patients experienced fatal adverse events (grade 5), both of which were unrelated to the PD-1 blockade.

**Conclusions:**

Patients with HAS can derive survival benefits from first-line treatment with PD-1 blockade combined with chemotherapy, and the treatment is well tolerated. Furthermore, this pathological subtype may serve as a predictive indicator of favorable efficacy for PD-1 blockade, regardless of the patients’ programmed death-ligand 1 combined positive score.

Implications for practice To date, no systematical studies based on the treatment of Hepatoid adenocarcinoma of the stomach (HAS) have been reported. As the first multicenter study to describe the efficacy and safety of PD-1 blockade plus chemotherapy in patients with HAS, this study provides valuable insights. PD-1 blockade plus chemotherapy demonstrated good efficacy and safety in patients with HAS. Hepatoid adenocarcinoma of the stomach, along with mismatch repair status and PD-L1 combined positive score, should each be considered as individual predictive markers for the efficacy of PD-1 blockade. For this group of patients, PD-1 blockade plus chemotherapy should be recommended as the standard first-line treatment.

## Introduction

Gastric cancer, one of the most common malignant tumors of the digestive system worldwide, continues to pose a significant threat to human health. According to the latest statistical data, the incidence of gastric cancer ranks fifth globally, while its mortality rate is the fourth highest.^1^ Due to the nonspecific early symptoms of gastric cancer, most patients are diagnosed at an advanced stage, resulting in a poor prognosis.^2^ The introduction of targeted therapy and immunotherapy has somewhat improved the prognosis for patients with advanced gastric cancer.^3^ Among these, human epidermal growth factor receptor 2 (HER2) is a critical therapeutic target in gastric cancer. An international multicenter phase III clinical trial (ToGA) demonstrated that the HER2 positivity rate in gastric cancer patients is 16.6%.^4^ Chemotherapy combined with anti-HER2 and programmed cell death protein 1 (PD-1) blockade has become the standard first-line treatment for patients with HER2-positive gastric cancer and programmed death-ligand 1 (PD-L1) combined positive score (CPS) ≥ 1.^5^ For patients with HER2-negative gastric cancer and PD-L1 CPS ≥ 5, chemotherapy combined with PD-1 blockade is the most commonly used clinical treatment option.^6^^,^^7^ In addition to the PD-L1 CPS, mismatch repair (MMR) status is the most recognized biomarker of PD-1 blockade efficacy, helping to identify the patient population most likely to benefit from such treatment.^8^ Despite these advancements, the 5-year survival rate for patients with advanced gastric cancer remains below 10%, highlighting the urgent need to identify predictive biomarkers for the efficacy of PD-1 blockade.

Hepatoid adenocarcinoma of the stomach (HAS) was first described by Ishikura et al. in 1985 as a type of gastric cancer characterized by elevated serum alpha-fetoprotein (AFP) levels and the presence of hepatocyte-like differentiation in tumor tissue histopathology.^9^ In 1993, Nagai et al. suggested that the defining feature of HAS is the appearance of hepatocyte-like differentiated areas in tumor tissue pathology, rather than AFP production,^10^ which contributed to a consensus on the histopathological diagnosis of HAS. This view was adopted by the World Health Organization in 2019, when HAS was officially recognized as a distinct subtype of gastric cancer.^11^

Hepatoid adenocarcinoma of the stomach, a rare subtype of gastric cancer, accounts for approximately 0.3% to 1.6% of all gastric cancers.^12^^,^^13^ Previous studies have shown that HAS is more prevalent in elderly individuals, with a higher incidence in men than in women.^14^ Hepatoid adenocarcinoma of the stomach predominantly occurs in the gastric antrum,^15^ often accompanied by elevated serum AFP levels, neural invasion, and metastasis to lymph nodes and the liver.^16^^,^^17^ Compared to common gastric adenocarcinoma, HAS exhibits a higher degree of malignancy and a poorer prognosis.^18^ Research on the treatment of HAS has primarily consisted of case reports, both domestically and internationally, with a lack of large clinical studies exploring standard treatment options. Previous case reports suggest that patients with HAS may benefit from PD-1 blockade.^19^^,^^20^ In this study, we retrospectively collected data from 25 patients with advanced HAS who received PD-1 blockade plus chemotherapy as the first-line treatment, further evaluating the efficacy and safety of the treatment modality in these patients. To the best of our knowledge, this represents the largest report based on the current sample size.

## Materials and methods

### Patient population

A total of 181 patients with pathologically confirmed HAS were retrospectively identified between January 2018 and January 2024 from 6 major medical centers. Among them, 25 patients who received first-line PD-1 blockade plus chemotherapy were included in this study from 4 centers, including the First Affiliated Hospital of Nanchang University, the Sun Yat-sen University Cancer Center, the Nanfang Hospital of Southern Medical University, and the First Affiliated Hospital of Soochow University. Patients were excluded if they met any of the following conditions: (1) PD-1 blockade was not used or not applied in the first-line treatment; (2) no measurable lesion; (3) incomplete follow-up data; (4) involvement of primary malignant tumors from other systems.

### Data collection and outcomes

This study aimed to retrospectively evaluate the efficacy and safety of first-line PD-1 blockade in patients with HAS. The patients’ clinical pathological data, their signs and symptoms, and laboratory test and imaging data were collected from electronic medical records. Radiological images of patients at each participating center were retrospectively reviewed by oncologists from the respective center. Imaging data included baseline and follow-up scans. Tumor responses were interpreted with reference to the RECIST version 1.1 criteria when applicable. The timing of imaging evaluations varied according to patients’ treatment regimens but generally followed institutional protocols, typically occurring every 6 weeks. Adverse events were retrospectively collected from medical records and summarized based on the original grading documented by physicians.

### Statistical analysis

Continuous variables were presented as medians with interquartile ranges (IQRs) or 95% confidence intervals (95% CIs), while categorical variables were expressed as frequencies with percentages. Progression-free survival (PFS) was defined as the time from the initiation of treatment to tumor progression or death from any cause. Overall survival (OS) was defined as the time from the initiation of treatment to death from any cause. The Kaplan-Meier method was employed to estimate PFS and OS distributions, with survival analysis performed using the Log-rank test. Statistical analysis was conducted using IBM SPSS Statistics 25, and graphing was done with R 4.1.3. A *P*-value of less than 0.05 was deemed statistically significant.

## Results

### Patient characteristics

**Figure 1.**
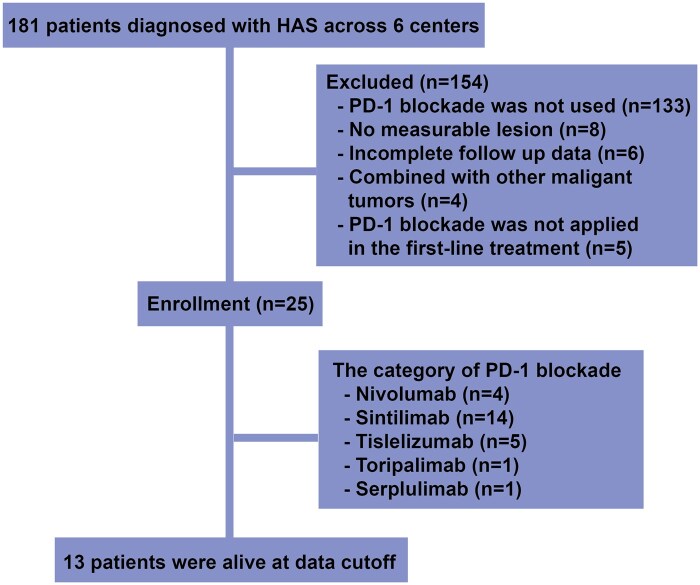
Flow diagram of this study.

This study included 25 patients with HAS who received first-line treatment with PD-1 blockade combined with chemotherapy (Figure 1). The clinical and pathological characteristics of these patients were summarized below (Table 1). The median age of the cohort was 61 years (range: 34-78), with the majority of patients being male (22, 88.0%). Thirteen patients (52.0%) had recurrence after radical gastrectomy for gastric cancer. Eleven patients (44.0%) had primary tumors located in the gastric antrum. Most patients had poorly differentiated gastric cancer (17, 68.0%). All 25 patients were mismatch repair protein stable. Eight patients (32.0%) had PD-L1 CPS <5, 8 patients (32.0%) had PD-L1 CPS ≥ 5, and 9 patients (36.0%) had unknown PD-L1 CPS. Before treatment, 15 patients (60.0%) had liver metastases. Thirteen patients (52.0%) had elevated AFP levels, and most patients (20, 80.0%) had normal carcinoembryonic antigen (CEA) levels. Seven patients (28.0%) were HER2 positive, of whom 5 (20.0%) received first-line treatment with trastuzumab combined with PD-1 blockade and chemotherapy. Among the 25 patients treated with PD-1 blockade, the drugs used included nivolumab (4, 16.0%), sintilimab (14, 56.0%), tislelizumab (5, 20.0%), toripalimab (1, 4.0%), and serplulimab (1, 4.0%). Regarding chemotherapy, 22 patients (88.0%) received a regimen primarily based on oxaliplatin, and 3 patients (12.0%) were treated with a regimen centered on taxanes.

**Table 1.** Patient characteristics.

Variables	ALL patients (*N* = 25)
**Age (years)**	
** Median, range**	61 (34-78)
**Gender (*n*, %)**	
** Male**	22 (88.0%)
** Female**	3 (12.0%)
**Primary tumor resection (*n*, %)**	
** Yes**	13 (52.0%)
** No**	12 (48.0%)
**Location (*n*, %)**	
** Cardia**	2 (8.0%)
** Fundus**	1 (4.0%)
** Body**	11 (44.0%)
** Antrum**	11 (44.0%)
**Liver metastasis**	
** Yes**	15 (60.0%)
** No**	10 (40.0%)
**Histological differentiation (*n*, %)**	
** Poorly**	17 (68.0%)
** Moderately poorly**	7 (28.0%)
** Moderately**	1 (4.0%)
**MMRstatus (*n*, %)**	
** pMMR**	25 (100.0%)
**PD-L1 CPS (*n*, %)**	
** <5**	8 (32.0%)
** ≥5**	8 (32.0%)
** NA**	9 (36.0%)
**AFP level (*n*, %)**	
** Normal**	8 (32.0%)
** High**	13 (52.0%)
** NA**	4 (16.0%)
**CEA level (*n*, %)**	
** Normal**	20 (80.0%)
** High**	5 (20.0%)
**HER2 status (*n*, %)**	
** Negative**	18 (72.0%)
** Positive**	7 (28.0%)
**Anti-HER2 therapy (*n*, %)**	
** Yes**	5 (20.0%)
** No**	20 (80.0%)
**Chemotherapy regimen (*n*, %)**	
** Based on oxaliplatin**	22 (88.0%)
** Based on taxanes**	3 (12.0%)
**Anti-PD-1 therapy type (*n*, %)**	
** Nivolumab**	4 (16.0%)
** Sintilimab**	14 (56.0%)
** Tislelizumab**	5 (20.0%)
** Toripalimab**	1 (4.0%)
** Serplulimab**	1 (4.0%)

Abbreviations: AFP, alpha-fetoprotein; CEA, carcinoembryonic antigen; HER2, human epidermal growth factor receptor 2; MMR, mismatch repair; pMMR, proficient mismatch repair; PD-1, programmed cell death protein 1; PD-L1 CPS, programmed death-ligand 1 combined positive score.

### Treatment responses and survival outcomes

Among the 25 patients with HAS receiving first-line treatment with PD-1 blockade combined with chemotherapy, the best response observed showed that a complete response (CR) was achieved in 2 patients, and a partial response (PR) was observed in 17 patients, resulting in an objective response rate (ORR) of 76.0%. Additionally, stable disease was observed in 3 patients, leading to a disease control rate (DCR) of 88.0% (Table 2). Table S1 further presents the treatment outcomes stratified by therapeutic regimen.

**Table 2.** Response outcome.

Response	*N* (%)
**CR**	2 (8.9%)
**PR**	17 (68.0%)
**SD**	3 (12.0%)
**PD**	3 (12.0%)
**ORR (CR+PR)**	19 (76.0%)
**DCR (CR+PR+SD)**	22 (88.0%)

Abbreviations: CR, control rate; DCR, disease control rate; PR, partial response; PD, progressive disease; SD, stable disease; ORR, objective response rate.

**Figure 2.**
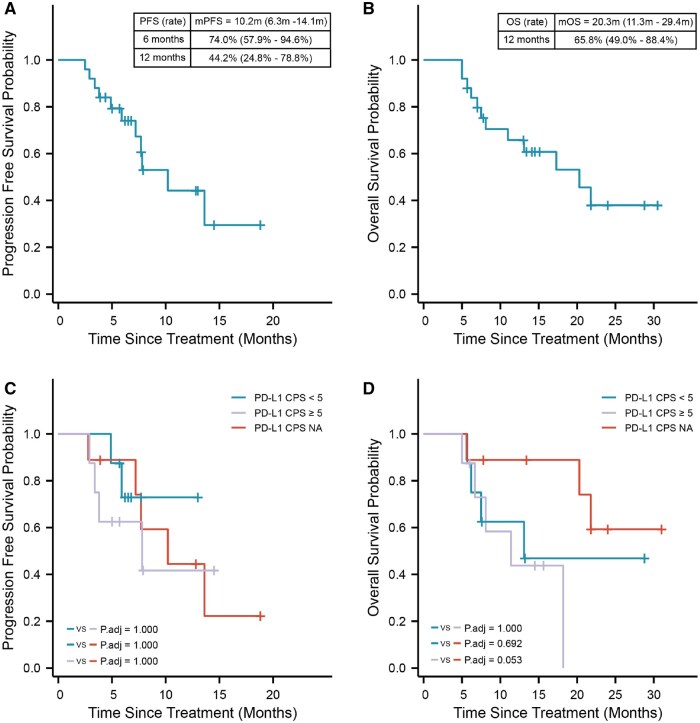
Patient’s survival curve distribution map. (A) A Kaplan-Meier estimate of progression-free survival in all patients. (B) A Kaplan-Meier estimate of overall survival in all patients. (C) Subgroup analysis of progression-free survival according to PD-L1 CPS. (D) Subgroup analysis of overall survival according to PD-L1 CPS. CPS, combined positive score.

The follow-up cutoff date was August 2024, with a median follow-up duration of 13.1 months. As of the cutoff, the mPFS for the 25 patients was 10.2 months (95% CI, 6.3-14.1), and the mOS was 20.3 months (95% CI, 11.3-29.4). The 6-month and 12-month PFS rates were 74.0% (95% CI, 57.9%-94.6%) and 44.2% (95% CI, 24.8%-78.8%), respectively, while the 12-month OS rates were 65.8% (95% CI, 49.0%-88.4%) (Figure 2A and B). Additionally, to further investigate the impact of PD-L1 CPS on the prognosis of patients with HAS, patients were categorized into 3 groups: PD-L1 CPS <5, PD-L1 CPS ≥5, and PD-L1 CPS unknown. The analysis revealed no statistically significant difference in PFS and OS among the 3 groups (Figure 2C and D), further confirming that PD-1 blockade demonstrates good efficacy in patients with HAS regardless of their PD-L1 CPS.

### Safety

Treatment-related adverse events of various grades were observed in 20 patients (80.0%) (Table 3). The most common adverse reactions were white blood cell (WBC) count decreased (12, 48.0%), followed by decreased neutrophil count (9, 36.0%), decreased platelet count (7, 28.0%), anemia (7, 28.0%), nausea (7, 28.0%), and peripheral neuropathy (6, 24.0%). The majority of adverse reactions were grade 1-2, with only a few cases of grade 3-4 reactions, and 2 grade 5 reactions were observed. Both patients who experienced grade 5 reactions died from severe pneumonia, subsequent examination revealed that one patient was diagnosed with COVID-19 and the other with bacterial pneumonia, neither of which was related to the PD-1 blockade. Both patients were evaluated as PR, and no tumor progression was observed at the time of death. One patient developed grade 4 immune-related enteritis during immunotherapy and discontinued treatment; this patient was evaluated as PR. Another patient developed chest tightness and elevated creatine kinase, indicative of immune-related grade 2 myocarditis.

**Table 3.** Treatment-related adverse events.

Adverse events	Grade 1-2 *N* (%)	Grade 3-4 *N* (%)	Grade 5 *N* (%)
**WBC count decreased**	10 (40.0%)	2 (8.0%)	0
**Neutrophil count decreased**	6 (24.0%)	3 (12.0%)	0
**Platelet count decreased**	4 (16.0%)	3 (12.0%)	0
**Anemia**	2 (8.0%)	5 (20.0%)	0
**Nausea**	7 (28.0%)	0	0
**Peripheral neuropathy**	6 (24.0%)	0	0
**Vomiting**	5 (20.0%)	0	0
**Diarrhea**	4 (16.0%)	1 (4.0%)	0
**Mucosal reaction**	3 (12.0%)	0	0
**Hypothyreosis**	3 (12.0%)	0	0
**Pneumonia**	0	0	2 (8.0%)
**ALT/AST** **increased**	2 (8.0%)	0	0
**Rash**	2 (8.0%)	0	0
**Immune enteritis**	0	1 (4.0%)	0
**Myocarditis**	1 (4.0%)	0	0

Abbreviation: ALT, alanine aminotransferase; AST, aspartate aminotransferase; WBC, white blood cell.

## Discussion

This retrospective, multicenter study included 25 patients with advanced HAS treated with first-line PD-1 blockade plus chemotherapy, representing the largest cohort reported to date. The clinical and pathological characteristics of the patients in this study are consistent with those reported in previous studies,^12^ with a high prevalence among middle-aged and elderly males. Gastric tumors predominantly arise in the gastric antrum and are frequently accompanied by distant liver metastases and elevated serum AFP levels. Additionally, a prior study suggested that HER2 is often highly expressed in gastric cancer with hepatoid adenocarcinoma differentiation.^21^ In this study, the proportion of patients with HER2-positive HAS was 28.0%, which is significantly higher than the HER2 positivity rate observed in the ToGA study of patients with conventional gastric adenocarcinoma.^4^

Hepatoid adenocarcinoma of the stomach, a rare pathological subtype of gastric cancer, is characterized by strong invasiveness, high malignancy, and poor prognosis, often accompanied by liver metastasis and elevated serum AFP levels. It shares similar clinical and pathological features with AFP-producing gastric cancer.^22^ A phase II, multi-center, single-arm clinical study (NCT04609176) enrolled 36 patients with stage III-IV unresectable or metastatic gastric or gastroesophageal junction adenocarcinoma, whose AFP levels were more than twice the upper normal limit. These patients were treated with apatinib combined with camrelizumab and the SOX regimen. Among the 35 evaluable patients, the ORR and DCR were 55.6% and 86.1%, with 12-month PFS and OS rates of 42.1% and 63.7%, respectively, confirming that AFP-producing gastric cancer may benefit from PD-1 blockade. However, due to the low incidence of HAS, no standard treatment regimen exists. Since HAS shares similar clinical and pathological features with AFP-producing gastric cancer, and prior reports have suggested the favorable efficacy of PD-1 blockade in HAS, this study aimed to evaluate the response of HAS to immunotherapy.^19^^,^^20^^,^^23^

The CheckMate-649 study demonstrated that patients with positive PD-L1 expression may benefit from the combination of nivolumab and chemotherapy, establishing the foundation for using immunotherapy combined with chemotherapy as a first-line treatment for gastric cancer.^24^ The publication of subsequent clinical research data, including ORIENT-16,^6^ RATIONALE-305,^25^ GEMSTONE-303,^26^ and KEYNOTE-859^27^ further solidifies the role of PD-1 blockade combined with chemotherapy as a first-line treatment for HER2-negative advanced gastric cancer patients (Table 4). In studies such as CheckMATE 649 and Orient-16, no survival benefit was observed in the PD-1 blockade combined with chemotherapy group compared to the chemotherapy-only group in patients with PD-L1 CPS < 5. Therefore, the National Comprehensive Cancer Network (NCCN) guidelines recommend nivolumab or sintilimab combined with chemotherapy as a category 1 recommendation for patients with PD-L1 CPS ≥ 5.

**Table 4.** Summary table of the efficacy of PD-1 blockade plus chemotherapy or dual immunotherapy as first-line treatment for gastric cancer in clinical trials.

	CheckMATE-649		ORIENT-16		GEMSTONE-303	RATIONALE-305		KEYNOTE-859		NO LIMIT	NCT04609176	This study
Group	ALL	CPS ≥ 5	ALL	CPS ≥ 5	CPS ≥ 5	ALL	TAP ≥ 5	ALL	CPS ≥ 10	ALL (MSI-H)	ALL (AFP positive)	ALL
**ORR (%)**	58	60	58.2	63.6	68.6	47.3	50.4	51.3	60.1	62.1	55.6	76.0
**DCR (%)**	NA	NA	87.7	90.1	NA	89.8	88.3	NA	NA	79.3	86.1	88.0
**mOS (months)**	13.8	14.4	15.2	18.4	17.81	15	17.2	12.9	15.7	NA	NA	20.3
**mPFS (months)**	7.7	7.7	7.1	7.7	7.62	6.9	7.2	6.9	8.1	13.8	NA	10.2
**12-month OS rate (%)**	55	57	NA	NA	61.25	57.9	59.8	53	61	80	63.7	65.8
**12-month PFS rate (%)**	33	37	NA	NA	NA	30.7	33.8	29	37	73	42.1	44.2

Abbreviations: AFP, alpha-fetoprotein; CPS, combined positive score; DCR, disease control rate; mOS, median overall survival; mPFS, median progression-free survival; ORR, objective response rate.

In this study, the median follow-up time for the 25 patients was 13.1 months, with an ORR of 76.0% and a DCR of 88.0%. The median PFS and OS were 10.2 months (95% CI, 6.3-14.1) and 20.3 months (s, 11.3-29.4), respectively. These results are superior to those reported in previous studies on first-line treatment with PD-1 blockade combined with chemotherapy for gastric adenocarcinoma patients. Furthermore, stratification by PD-L1 CPS revealed no statistically significant survival differences. These findings suggest that HAS, as a pathological subtype, may serve as an independent predictive indicator for the efficacy of PD-1 blockade, alongside PD-L1 CPS and mismatch repair status.

In this study, 20 patients experienced adverse reactions of varying degrees. The most common adverse reactions included WBC count decreased, neutrophil count decreased, platelet count decreased, anemia, nausea, and vomiting. Three patients developed immune-related hypothyroidism. One patient developed immune-related colitis during immunotherapy, resulting in the discontinuation of treatment, with a treatment evaluation of PR, and the disease progressed shortly after discontinuation of PD-1 blockade. Another patient discontinued PD-1 blockade due to suspected immune-related myocarditis, and this patient’s treatment evaluation was also PR. Two patients died due to non-tumor-related causes, one was diagnosed with COVID-19, and the other with bacterial pneumonia. Both patients had been receiving first-line PD-1 blockade combined with chemotherapy, with treatment evaluations of PR, and no progression was observed on imaging prior to their deaths. The OS of these 2 patients was 5.7 months and 6.2 months, respectively. These findings demonstrate that patients with HAS can tolerate PD-1 blockade combined with chemotherapy well.

This study has several limitations, including its retrospective nature and relatively short follow-up period. Although the study included multiple centers, the sample size remains small, and all patients were from China, which may lead to bias in the subgroup analysis. Additionally, the types of PD-1 blockade used and the chemotherapy regimens administered varied among patients. Furthermore, the study lacks a control group. As a retrospective study, this research may have missed certain adverse reactions. The large-scale, multicenter, prospective phase III clinical trials are expected to further confirm the efficacy and safety of PD-1 blockade for patients with HAS in the future.

## Conclusions

Hepatoid adenocarcinoma of the stomach, a rare gastric cancer subtype, may benefit from first-line PD-1 blockade combined with chemotherapy, with good tolerance. Regardless of PD-L1 CPS, this treatment demonstrates favorable efficacy in patients with HAS. Thus, HAS may serve as a predictive indicator for the efficacy of PD-1 blockade.

## Supplementary Material

oyaf312_Supplementary_Data

## Data Availability

The original data showed in this study are available from the corresponding authors upon reasonable request.
