# Supplementary material for: A multicenter retrospective study of PD-1 blockade plus chemotherapy as first-line therapy in advanced hepatoid adenocarcinoma of the stomach
Source: Oncologist. 2025 Sep 25;30(10):oyaf312. doi: 10.1093/oncolo/oyaf312 (PMC12573259; doi:10.1093/oncolo/oyaf312)
Supplement: oyaf312_Supplementary_Data [file oyaf312_supplementary_data.zip › Supplemental Table S1.docx]

Supplemental Table S1: Response outcome Stratified by Treatment Group

| Group | N | CR | PR | SD | PD | ORR (%) | DCR (%) |
| --- | --- | --- | --- | --- | --- | --- | --- |
| PD-1 blockade plus oxaliplatin-based chemotherapy | 17 | 2 | 11 | 2 | 2 | 76.5% | 88.2% |
| PD-1 blockade plus paclitaxel-based chemotherapy | 3 | 0 | 2 | 0 | 1 | 66.70% | 66.70% |
| PD-1 blockade plus oxaliplatin-based chemotherapy and anti-HER2 | 5 | 0 | 4 | 1 | 0 | 80% | 100% |
